# Endovascular external carotid artery occlusion for brain selective targeting: a cerebrovascular swine model

**DOI:** 10.1186/s13104-015-1714-7

**Published:** 2015-12-21

**Authors:** Sundeep Mangla, Jae H. Choi, Frank C. Barone, Carol Novotney, Jenny Libien, Erwin Lin, John Pile-Spellman

**Affiliations:** Division of Interventional Neuroradiology, SUNY Downstate Medical Center, 470 Clarkson Ave, B7525, Brooklyn, NY 11203 USA; Department of Neurology, SUNY Downstate Medical Center, Brooklyn, NY USA; Hybernia Medical, LLC, Pelham, NY USA; Division of Comparative Medicine, SUNY Downstate Medical Center, Brooklyn, NY USA; Department of Pathology, SUNY Downstate Medical Center, Brooklyn, NY USA; Department of Radiology, Brigham and Women’s Hospital, Boston, MA USA; Neurological Surgery, PC, Lake Success, NY USA

**Keywords:** Animal research, Cerebrovascular, Endovascular intervention, Selective cooling, Swine model

## Abstract

**Background:**

The choice of an animal model for cerebrovascular research is often determined by the disease subtype to be studied (e.g. ischemic stroke, hemorrhage, trauma), as well as the nature of the intervention to be tested (i.e. medical device or pharmaceutical). Many initial studies are performed in smaller animals, as they are cost-effective and their encephalic vasculature closely models that of humans. Non-human primates are also utilized when confirmation or validation is required on higher levels and to test larger devices. However, working with primates is complex and expensive. Intermediate sized animal models, such as swine and sheep, may represent a valuable compromise. Their cerebrovascular anatomy, however, comes with challenges because of the natural higher external carotid artery perfusion and the existence of a rete mirabile. We describe a modification to the traditional swine cerebrovascular model that significantly enhances selective brain hemispheric perfusion, limiting external carotid perfusion and dilution.

**Results:**

We investigated whether unilateral endovascular coil-embolization of external carotid artery branches in swine would lead to increased brain perfusion, altering cerebral circulation so that it more closely models human cerebral circulation. Equal amounts of approximately 4 °C cold saline were injected in 6 Yorkshire pigs into the ipsilateral common carotid artery before and after embolization. Hemispheric temperature changes from pre- and post-embolization were obtained as a measure of brain perfusion and averaged and compared using non-parametric statistical tests (Wilcoxon signed rank test, Mann–Whitney U Test). Graphs were plotted with absolute changes in hemispheric temperature over time to determine peak temperature drop (PTD) and corresponding time to peak (TTP) following the cold bolus injection. There was a 288 ± 90 % increase in ipsilateral brain cooling after embolization indicating improved selective blood flow to the brain due to this vascular modification.

**Conclusion:**

We have developed an effective, selective vascular brain model in swine that may be useful as a practical and cost-reducing intermediate step for evaluating target dose–responses for central nervous system drugs and brain selective interventions, such as local hypothermia.

**Electronic supplementary material:**

The online version of this article (doi:10.1186/s13104-015-1714-7) contains supplementary material, which is available to authorized users.

## Background

Research involving the cerebrovascular system has continued to accelerate, as has the need for innovative animal models to test novel devices and therapeutic strategies. The choice of an animal model is determined by a variety of factors, including the disease subtype to be studied (e.g. ischemic stroke, hemorrhage, trauma), as well as the nature of the intervention (i.e. medical device or pharmaceutical) to be tested [1]. Many initial studies are performed in smaller animals (mice, rats, rabbits), as they are cost-effective and their encephalic vasculature closely models that of humans. Non-human primates (e.g. Baboons, Macaques) are also utilized when confirmation is required (i.e. to test larger devices or to improve human validation). The increased complexity and expense of working with primates, however, limits the capacity of many research teams to execute these higher level validations. Intermediate sized animal models, such as swine and sheep, may represent a valuable compromise for cerebrovascular research [2–5]. However, their pre-encephalic vasculature has been considered problematic because of their pre-cerebral rete that is bilaterally connected to the common carotid artery (CCA) by the ascending pharyngeal artery (AsPharA) [6, 7]. This rete supplies their Circle of Willis (CoW) through two small internal carotid arteries (ICA) that branch off of the rete. Furthermore, in animals the carotid system contributes proportionally more blood to the external carotid arteries (compared to humans) to supply their snout and relatively massive facial and masticatory muscles [8]. Here, we describe a modification to the traditional swine cerebrovascular model that significantly enhances selective brain hemispheric perfusion while limiting external carotid perfusion and dilution. We believe that this modification will enhance our ability to evaluate brain-selective therapeutics and diagnostics, and target dose-responses in this larger non-primate model, a model particularly well-suited for investigating larger endovascular devices and central nervous system drugs.

## Methodology

### Animal care

Procedures were conducted in accordance with all Federal and State animal welfare laws, regulations, policies and guideline, in compliance with the ARRIVE guidelines, and with approval by the SUNY Downstate Medical Center Institutional Animal Care and Use Committee. Six female white Yorkshire swine (35–55 kg, ages 18–36 weeks), were used in this study. Free access to food and water was given until the night before the procedure, at which time only water was allowed. Sedation was induced by atropine 0.04 mg/kg IM and Telazol 2–5 mg/kg IM. General anesthesia was induced with inhalant isoflurane (3–5 %) by mask with 100 % oxygen at 3–5 L/min and maintained by 1–3 % isoflurane inhalant. To provide balanced anesthesia/analgesia isoflurane was combined with Fentanyl at 50 µg/kg initial bolus followed by continuous administration at 30–100 µg/kg IV. Hemodynamic monitoring (heart rate, systolic and diastolic blood pressure) and expired air CO_2_ monitoring were performed continuously. Endotracheal intubation was performed via tracheotomy. Animals were ventilated at respiratory rate 10–18/min, tidal volume 10–15 ml/kg, inspiratory/expiratory ratio 1:3–1:4, peak inspiratory pressure 15–20 cmH_2_O, end-tidal CO2 35–45 mmHg, positive end expiratory pressure 3–5 cmH_2_O, and sigh every 15 min to 25–30 cmH_2_O. Two intravenous lines were placed within bilateral ear veins for IV access (replacement fluid Normosol-R 5–10 ml/kg/hr). An arterial line was placed in either the contralateral hind or fore limb to obtain continuous pressure recordings and intermittent blood sampling. Systemic temperature probe was placed within the esophagus or rectum to monitor body core temperature and to prevent body cooling using a circulating warming blanket. A urinary catheter was placed to monitor output. After stable surgical anesthesia was achieved Vecuronium (Norcuron) was administered with a loading dose of 0.05–0.1 mg/kg and maintained at 0.8–1.2 mcg/kg/min. After the experiments the animals were euthanized with an intravenous injection of 160 mg/kg of Fatal Plus (Pentobarbital). Finally, the brain was extracted, fixed in formalin, and evaluated for ischemic damage by gross and histopathologic (H&E stain) examination.

### Intracranial probe placement

The animals were placed prone, and surgical dissection of the cranium was performed from immediately anterior to the ears to approximately 7–10 cm anterior to the orbits. The bregma was located and burr holes were created 2 cm cephalad and 1.5–2.0 cm lateral to the sagittal suture bilaterally with the Raumedic (Helmbrechts, Germany) manual drill system. The dura was pierced bilaterally and the intracranial probes for temperature and intracranial pressure (ICP) (Neurovent, Raumedic) were advanced approximately 3.5–4.5 cm into the cranial vault and frontal lobes from the osseous surface and secured. Probe positioning was confirmed with appropriate physiologic measurements for expected ICP and temperature on the Raumedic monitors.

### Endovascular vessel modification and vessel blood flow measurements

Each animal underwent surgical dissection of superficial fascial and muscular planes for an ultrasound-guided groin approach of the common femoral artery. Access was established using a micropuncture needle and wire, with serial dilatation and placement of a 7-French 15 cm sheath (Pinnacle, Terumo, NJ) that was applied to a continuous heparinized flush solution. After vascular access and intracranial probe placement 4000 IU Heparin was administered IV as a bolus with additional 2000 IU/h for the duration of the study procedure. Ultrasound studies of bilateral CCAs were performed to assess maximum diameter (Dmax, intima to intima) and peak systolic velocity (psFV at 60° angle with cursor parallel to vessel wall) in 3 animals (4–8 MHz B-Mode/Doppler, SonoSite MicroMaxx, UDS, CA, USA). A hypothetical maximum CCA blood flow (BFc) was estimated with the following equation: ${\text{BFc}} = \pi *\left( {{\text{Dmax}}/ 2} \right)^{ 2} *{\text{psFV}}$. Utilizing a single plane fluoroscopic C-arm with Digital Subtraction Angiography (DSA) (OEC 9800, GE Healthcare, Little Chalfont, UK), a 5-French 45 degree tip endovascular catheter was advanced over a 0.035 inch hydrophilic guidewire and selective catheterization of one CCA was performed. Antero-posterior projection DSA of the CCA and cranial vasculature was performed using 8–9 ml of iodinated contrast (Omnipaque 300, GE Healthcare, OH, USA), demonstrating the AsPharA, rete, CoW, and external carotid arterial (ECA) branches at baseline. Baseline temperature and ICP readings were recorded bilaterally. A rapid bolus of cold saline (10 ml/3 s, 4 °C) was infused once or two times in rapid succession into the ipsilateral CCA, and continuous readings of temperature and ICP were recorded. Cold Saline was utilized as a diffusible tracer, similar to Xenon 133, as opposed to Iodinated Contrast, which represents a non-diffusible tracer.

**Fig. 1:**
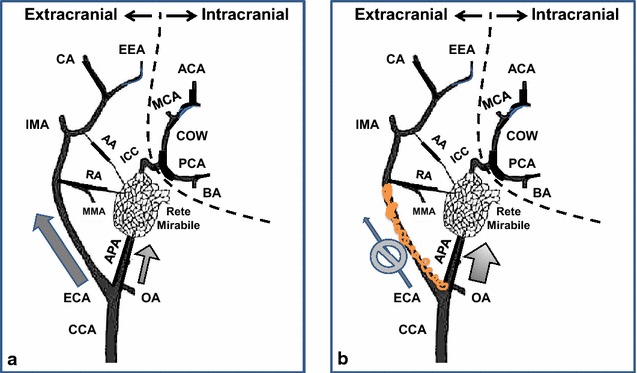
Vessel modification and hemodynamic changes. **a** Baseline, right common carotid artery (CCA). **b** Decrease in blood flow in the external carotid artery (ECA) and increase in the ascending pharyngeal artery (APA) following partial embolization of ECA branches. *CC* common carotid artery, *O* occipital artery, *EC* external carotid artery, *AP* ascending pharyngeal artery. Branches of the external ethmoidal artery, (EE): *MM* middle meningeal artery, *RA* ramus anastomoticus, *AA* arteria anastomotica, *IM* internal maxillary artery, *C* ciliary artery. Intracranial anatomy: *PC* posterior cerebral artery, *BA* basilar artery, *CW* circle of Willis, *MC* middle cerebral artery, *AC* anterior cerebral artery

The 5-French endovascular catheter was positioned beyond the origin of the ascending pharyngeal artery. Major branches of the ECA were identified (lingual and facial arteries) and selectively embolized with 0.035 inch stainless steel coils (Nester, Cook Medical, IN, USA). Control DSA was performed to demonstrate AsPharA, rete, CoW, and to visually confirm a significant reduction in contrast opacification of ECA branches (Fig. 1a/b). Follow-up ultrasound of both CCAs was performed. A rapid bolus of cold saline of equal amount as at baseline (10 ml/3 s, 4 °C) was infused into the ipsilateral CCA, and continuous readings of temperature and ICP were recorded.

Intracranial parameters and ultrasound data from pre- and post-embolization were averaged and compared using non-parametric statistical tests (Wilcoxon signed rank test, Mann–Whitney U Test). Graphs were plotted with absolute changes in hemispheric temperature (Celsius) over time (seconds) to determine peak temperature drop (PTD) and corresponding time to peak (TTP) following the cold bolus injection, pre and post ECA embolization. Statistical analyses were performed with SPSS software (version 15, SPSS, IL, USA).

## Results

**Fig. 2:**
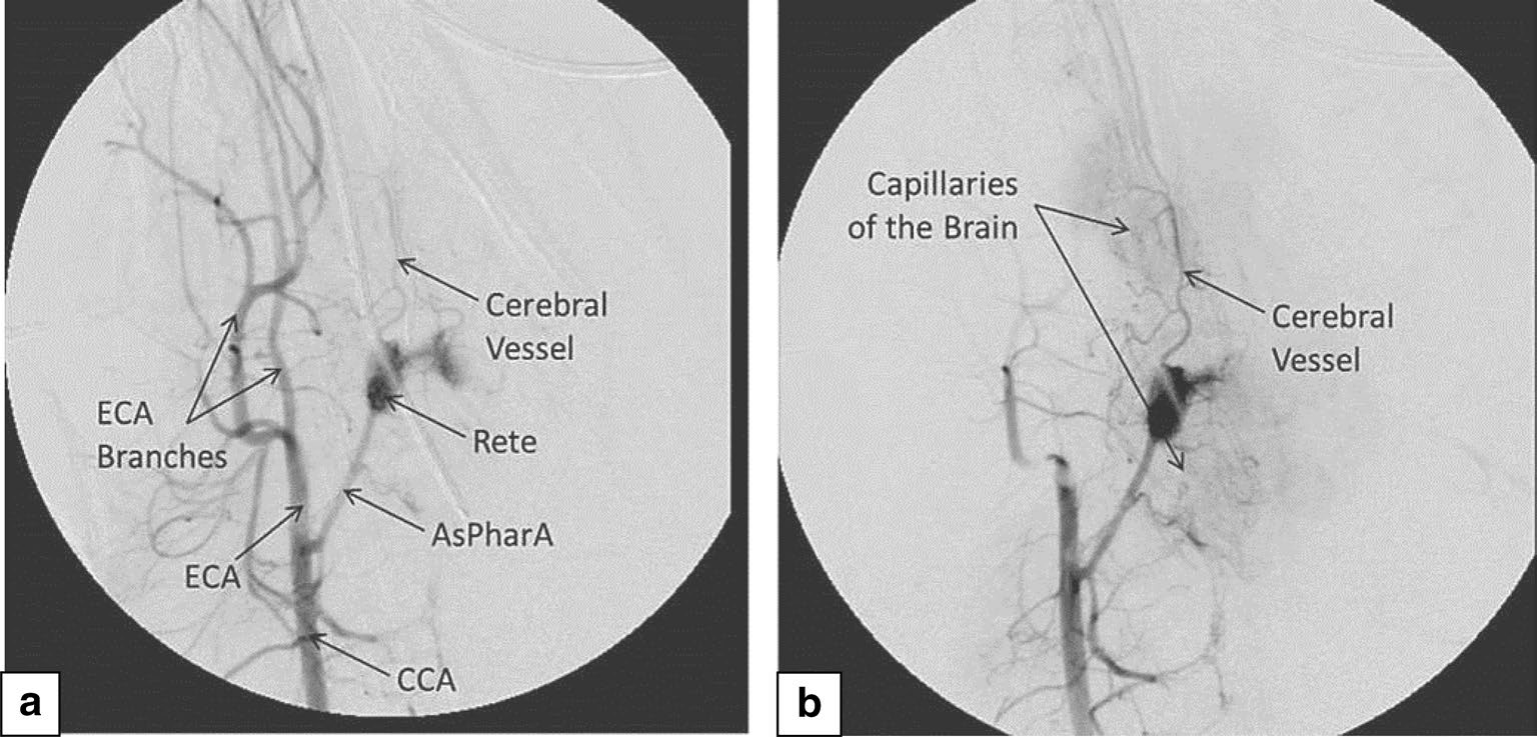
Cerebral angiogram in a representative animal after injection of contrast into the right CCA before (**a**) and after (**b**) embolization of ECA branches

Post-embolization of ECA branches, there was a significant reduction in contrast opacification of the ECA and its branches and significant enhancement of opacification of the AsPharA, rete, and CoW (Fig. 2a/b).

Correct placement of intracranial probes was confirmed: brain temperature right vs. left (37.2 ± 0.7 vs. 37.3 ± 0.8 °C; not significant) and ICP right vs. left (20.9 ± 4.3 vs. 21.4 ± 4.5 mmHg; n.s.).

At baseline, the average peak decrease in ipsilateral brain temperature (PTD) following the cold bolus was 0.22 ± 0.12 °C, whereas after the embolization it was 0.66 ± 0.50 °C (p = 0.028) resulting in an average increase of 288 ± 90 % of selective brain cooling. The respective changes in the contralateral hemisphere (baseline 0.10 ± 0.05 °C, post-embolization 0.36 ± 0.26 °C; p = 0.046) were in the range of 43–54 % of the changes seen on the ipsilateral side (Additional file 1).

**Fig. 3:**
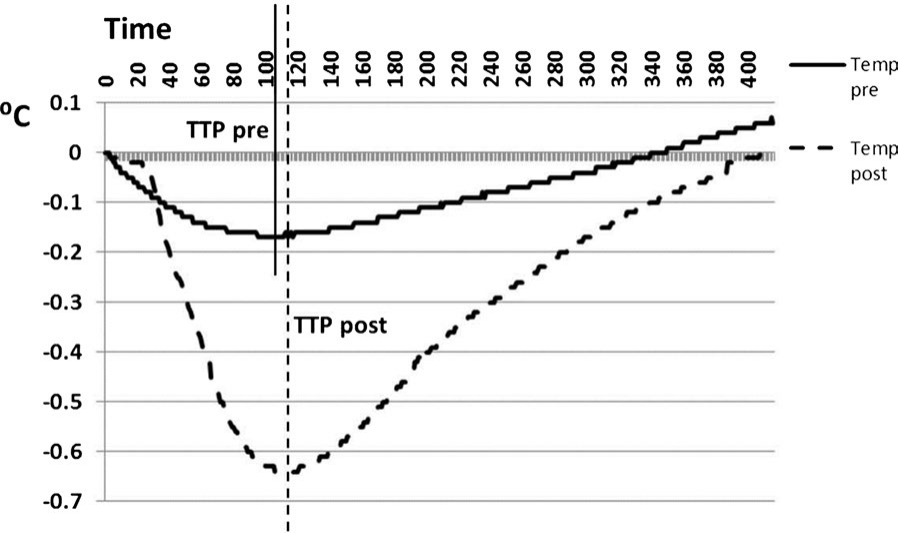
Hemispheric temperature change and time to peak (TTP) following cold saline injection in a representative animal before (Temp pre) and after (Temp post) vascular modification. Time in seconds

The average TTP was similar between the ipsilateral hemisphere versus contralateral before (98 ± 68 versus 102 ± 50 s; p = 0.818) and after (124 ± 133 versus 146 ± 179 s; p = 1.000) the embolization (example in Fig. 3). TTP was similar between pre- and post-embolization within the same hemispheres (right p = 0.600, left p = 0.600).

ICP remained unchanged from baseline values following the cold bolus injections (22.1 ± 3.6–22.6 ± 3.6 mmHg; p = 0.236) in the ipsilateral hemisphere, whereas it increased slightly on the contralateral side (19.4 ± 4.7–20.1 ± 4.9 mmHg; p = 0.027).

CCA diameter (4.9 ± 0.4–4.4 ± 0.3 mm; p = 0.109) and psFV (107 ± 31–80 ± 40 cm/sec; p = 0.102) remained unchanged ipsilateral to the embolization. So did CCA diameter (48.3 ± 2.1–49.7 ± 1.2 mm; p = 0.285) and psFV (93 ± 23–137 ± 25 cm/sec; p = 0.109) on the contralateral side. The average (theoretical maximum) BFc for pre- and post-embolization was 1199 ± 225 and 753 ± 416 ml/min (p = 0.109) for the right CCA and 1032 ± 291 and 1590 ± 303 ml/min (p = 0.109) for the left CCA, respectively. BFc for both CCAs combined was similar before and after the embolization (2232 ± 515 versus 2344 ± 450 ml/min; p = 0.109).

The duration of the study, including preparation of the animals and the main procedure was 5–6 h. All animals were normothermic. Macro- and histopathology of the brain showed traumatic changes from the intracerebral probe placement and did not reveal any signs of ischemic damage.

## Discussion

Selective embolization of ECA vessels enhances the selective distribution of the tracer/indicator (cold bolus in this case) into the cerebral circulation without affecting cerebral perfusion or dynamics, effectively converting the swine CCA into a model of the ICA similar to humans and primates, albeit with an intervening rete. In our swine model embolization of ECA branches enhances selectivity for cerebral perfusion by approximately threefold as measured by PTD. Similar to humans, the ICA is the major contributor to cerebral perfusion in pigs, i.e. equivalent to AsPharA in pigs [7–9]. However, it is also known that in pigs there exist anastomoses between ECA and ICA territories (Fig. 1) that were not quantified in the present study [10]. Overall, our data suggest that no significant change in CBF occurred with the ECA embolization which is supported by similar TTP values and bulk CCA flows pre- and post-embolization. It should be noted that unlike traditional contrast-enhanced cerebral perfusion studies using CT or MRI, TTP (temperature drop) as measured in our study using a thermal diffusible tracer is not purely a function of vascular perfusion, but a function of heat-transfer between arterial blood and brain matter [11, 12]. Such heat-transfer is dependent on tissue perfusion, metabolism, and temperature differential [13]. As such, the cooling (wash-in) and rewarming (wash-out) phases have longer durations.

The main limitations for this model are secondary to the rete and its function as a barrier to creating an effective embolic model for focal stroke and its unknown physiologic role in cerebral autoregulation. Accurate measures for vessel and cerebral blood flow and ultrasound evaluations are needed to further validate its use for cerebrovascular studies.

Traditionally, swine models have been extensively used in pre-clinical studies involving functional genomics, organ transplant, atherosclerosis, trauma, and cardiovascular and neurological diseases [1]. The rete, although usually an obstacle to brain selective investigations, has also been utilized as a model for arteriovenous malformations [14]. However, in most cases the natural high ECA blood flow and the presence of a rete significantly impede studies of therapeutics selectively targeting the brain and may necessitate more invasive surgical means to achieve selectivity, such as vascular clamping and vessel reconstruction to create strokes, and the use of brain probes [1]. Proximal surgical ligation of the external carotid has been the traditional approach to creating a selective cerebrovascular model. This appears to be more reasonable when experimental procedures involve surgery in head and neck regions. The main advantage of the proposed endovascular approach includes minimizing surgical stress. This is particularly useful in experimental settings where brain-selective drugs and interventions are studied with endovascular means. In addition, more distal and extensive long-segment occlusions can be performed to minimize collateral diversion. Our endovascular vascular modification method may be helpful in reducing the need for more extensive measures, allow accurate and selective evaluation of dose-responses at the target organ via interventional testing of selective neuroprotective approaches, including delivery of pharmacologics, evaluation of medical devices, and application of selective brain cooling [15]. We believe our vascular modification will provide a much improved intervention dose-response sensitivity over traditional non-embolization models due to better cerebral-specific distribution in swine as occurs in primates.

In conclusion, the present results demonstrate enhanced selective blood flow to the brain ipsilateral to the vascular modification. This is a safe and practical method enabling brain-selective investigations of pharmacologic and interventional procedures in the pig.

## Availability and requirements

Project name: Hybernia Medical Brain Selective Targeting Project; Project home page: None; Operating system(s): Windows; Programming language: Proprietary; Other requirements: NI LabView, Raumedic Neurovent Datalogger; License: National Instruments, Raumedic; Any restrictions to use by non-academics: Proprietary.

## Availability of supporting data

The data set(s) supporting the results of this article is(are) included within the article (and its additional file(s)).

## Additional file

10.1186/s13104-015-1714-7 Temperature Data following cold saline injection at baseline and post modification.

## Abbreviations

AsPharA: ascending pharyngeal artery
CoW: Circle of Willis
CCA: common carotid artery
(DSA: digital subtraction angiography
ECA: external carotid arterial
ICA: internal carotid arteries
ICP: intracranial pressure
Dmax: maximum diameter
BFc: maximum CCA blood flow
psFV: peak systolic velocity
PTD: peak temperature drop
TTP: time to peak
